# Mechanical characterization of human versus porcine brain tissue under large strains

**DOI:** 10.1007/s10237-025-02040-8

**Published:** 2026-03-05

**Authors:** Nina Reiter, Sarah Nistler, Lucas Hoffmann, Lars Bräuer, Friedrich Paulsen, Silvia Budday

**Affiliations:** 1 Institute of Continuum Mechanics and Biomechanics, Friedrich-Alexander-Universität Erlangen-Nürnberg (FAU), Dr.-Mack-Str. 81, 90762 Fürth, Germany; 2 Department of Neuropathology, Universitätsklinikum Erlangen, Friedrich-Alexander-Universität Erlangen-Nürnberg (FAU), Schwabachanlage 6, 91054 Erlangen, Germany; 3 Institute of Functional and Clinical Anatomy, Friedrich-Alexander-Universität Erlangen-Nürnberg (FAU), Universitätsstr. 19, 91054 Erlangen, Germany

**Keywords:** Brain tissue, Human, Porcine, Mechanical testing, Viscoelasticity, Large strains, Compression, Tension, Shear

## Abstract

Due to the limited availability of human body donor brains, biomechanical testing for material parameter identification is often done on animal tissue. So far, only one study has compared human and animal brain tissue under indentation, which provides data for the small strain regime and hence is not applicable to large-strain scenarios like surgery. Here, we compare the large-strain behavior of human and porcine brain tissue from four anatomical regions (corona radiata, putamen, cerebellar white matter, and brain stem) under compression, tension, and shear. Our results from cyclic loading tests show that samples from the cerebral white matter behave similarly for both species under all loading modes, whereas samples from the cerebellar white matter are similar only under compression-tension, but not shear loading. For regions with mixed gray and white matter, we observe a softer behavior for porcine samples under all loading modes. The stress relaxation behavior is similar in both species, except for samples from the putamen. These findings indicate that porcine brain tissue can be used as an alternative for human tissue to study general tissue mechanics. However, since the porcine brain is much smaller and therefore, less brain regions can be characterized mechanically, human brain data are still needed to calibrate full brain simulation models.

**Supplementary Information:**

The online version contains supplementary material available at 10.1007/s10237-025-02040-8.

## Introduction

Finite element models of the brain are gaining importance, for instance to predict deformations that occur during surgical procedures and that lead to deviations between the actual and preoperatively determined position of regions of interest in the brain, which reduces the accuracy of neuronavigation systems (Hastreiter et al. 2004; Miller et al. 2019; Narasimhan et al. 2020). Compensating for interoperative deformations during surgery planning can improve surgical outcomes and minimize the risk of functional loss. These models require material parameters for brain tissue that cannot be obtained from in vivo experiments since the strains that are imposed during open brain surgeries exceed the strains that can be applied safely to living brains with techniques such as magnetic resonance elastography (Miller et al. 2019; Griffiths et al. 2024), where brain displacements are in the order of microns (McCracken et al. 2005; Sack et al. 2008). Therefore, simulations still have to rely on parameters obtained from ex vivo experiments on brain tissue.

Human brain tissue is considered the gold standard for such experiments, but its availability is very limited and there are only a few research groups that have published data from human tissue testing (Finan et al. 2017; Sundaresh et al. 2022; Chatelin et al. 2012; Budday et al. 2017; Forte et al. 2017; Hinrichsen et al. 2023; Menichetti et al. 2020). Human tissue for biomechanical research is usually obtained through brain surgeries (Finan et al. 2017; Sundaresh et al. 2022), autopsies (Chatelin et al. 2012; Budday et al. 2017), or tissue and body donations (Forte et al. 2017; Hinrichsen et al. 2023; Menichetti et al. 2020; Mallory et al. 2024). Surgical tissue can be obtained from surgeries that aim to extract focal epileptic lesions or tumors. While the amount of resected tissue is kept as small as possible, healthy tissue (so called access tissue) may need to be extracted when the lesion is located deeply in the brain (Straehle et al. 2023). A significant advantage of access tissue is that it can be tested directly after excision. A disadvantage is that only tissue from the cortex and subcortical white matter can be tested. Through autopsies or tissue donations, it is possible to obtain larger tissue pieces including a larger variety of brain regions or, in the case of body donations, the entire brain. However, tissue from autopsies or donations that is eligible for biomechanical testing usually becomes available only hours or days postmortem due to documentation requirements. Additional challenges are associated with the experimental process itself. To obtain material parameters that represent the behavior of the living brain, it is necessary to test fresh brain tissue that has not undergone any preservation procedure, such as (formalin) fixation or freezing, to not alter its mechanical behavior (Metz et al. 1970). Working with unfixed human tissue is challenging due to its soft nature (Budday et al. 2019) and requires biological safety standards that cannot be met in pure mechanics laboratories. In addition, brain tissue decomposes within days after death (Krassner et al. 2023) in a highly temperature dependent manner (Parisi et al. 2024), which imposes strict cooling requirements and limits the available time for performing experiments.

To circumvent these difficulties, many research groups have used animal tissue, for example pig or sheep brains obtained from local butchers, as a surrogate for human tissue (Faber et al. 2022). Pig brains share similarities with human brains and are therefore assumed to be a suitable alternative for human brain tissue for medical training (Sauleau et al. 2009), but so far there has been only one study that directly compared the mechanical behavior of human and porcine brain tissue under loading rates relevant to brain injury using the same testing setup and experimental conditions for both species (MacManus et al. 2020). In this study, human, pig, rat, and mouse brain samples were subjected to indentation tests and it was found that the indentation response of pig and mouse brains was most similar to the human brain in the investigated regions, i.e., the cortex, cerebellum, medulla oblongata, and pons, while the rat brain was the most significantly different among the compared surrogates.

However, insights gained from indentation testing are not necessarily transferrable to large-strain mechanical testing of brain tissue since indentation data cannot represent all loading modes (Budday et al. 2019), and drainage conditions differ in indentation versus multimodal testing (Greiner et al. 2021). It is thus still unclear whether the human and porcine brain also behave sufficiently similar under larger deformations to justify the use of porcine tissue for the calibration of material parameters for full brain modeling applications such as the simulation of surgical procedures.

Here, we compare the large-strain mechanical behavior of postmortem human and porcine brain tissue from four anatomical regions (corona radiata, putamen, cerebellar white matter, brain stem) using the same testing setup for both species. To comprehensively characterize the viscoelastic tissue behavior of both species, we subject all samples to a sequence of cyclic compression and tension, cyclic torsional shear, as well as compression, tension, and shear relaxation tests.

## Methods

### Human brains

We obtained 13 whole human brains from three female and ten male body donors (age range 62–92 years, further details given in Reiter et al. (2025)) who had given their written consent to donate their body to research. This study was approved by the Ethics Committee of Friedrich-Alexander-Universität Erlangen–Nürnberg, Germany, with the approval number 405_18 B. The data for white matter, cerebellum, and brain stem regions of the human brain were previously published in Reiter et al. (2025). However, here, one male donor brain was excluded from the study after histopathological analyses revealed significant Alzheimer’s-disease associated microstructural alterations. Further, we excluded samples that had heights outside the range of 4–6 mm to reduce the effects of strain rate dependence on the interpretability of the results, as further explained in Sect. 2.4.

We used brains of body donors that we could receive 24 h postmortem at the latest. We transported the brain to the biomechanics laboratory in a bucket that was either filled with artificial cerebrospinal fluid (ACSF), or, if ACSF was not available on short notice, with Ringer’s solution or phosphate-buffered saline solution (PBS) to keep the brain protected during transport (details on the fluids used for transport are given in Reiter et al. (2025)). We removed the dura mater and separated the brain stem and cerebellum from the cerebrum to facilitate cutting. Subsequently, we cut the cerebrum and cerebellum into 1–3-cm-thick coronal slices. We then moved the coronal slices and the brain stem into airtight containers that we filled with the fluid the brain had arrived in. There was usually enough fluid to almost fully immerse the slices. The boxes were kept refrigerated at $4\,^{\circ }\hbox {C}$ and only taken out of the refrigerator when a new sample was prepared. The mechanical experiments were completed within 98 h postmortem, with the postmortem interval defined as the time difference between the time of death specified in the donor’s death certificate and the time at which the experiment was started.

### Porcine brains

**Fig. 1:**
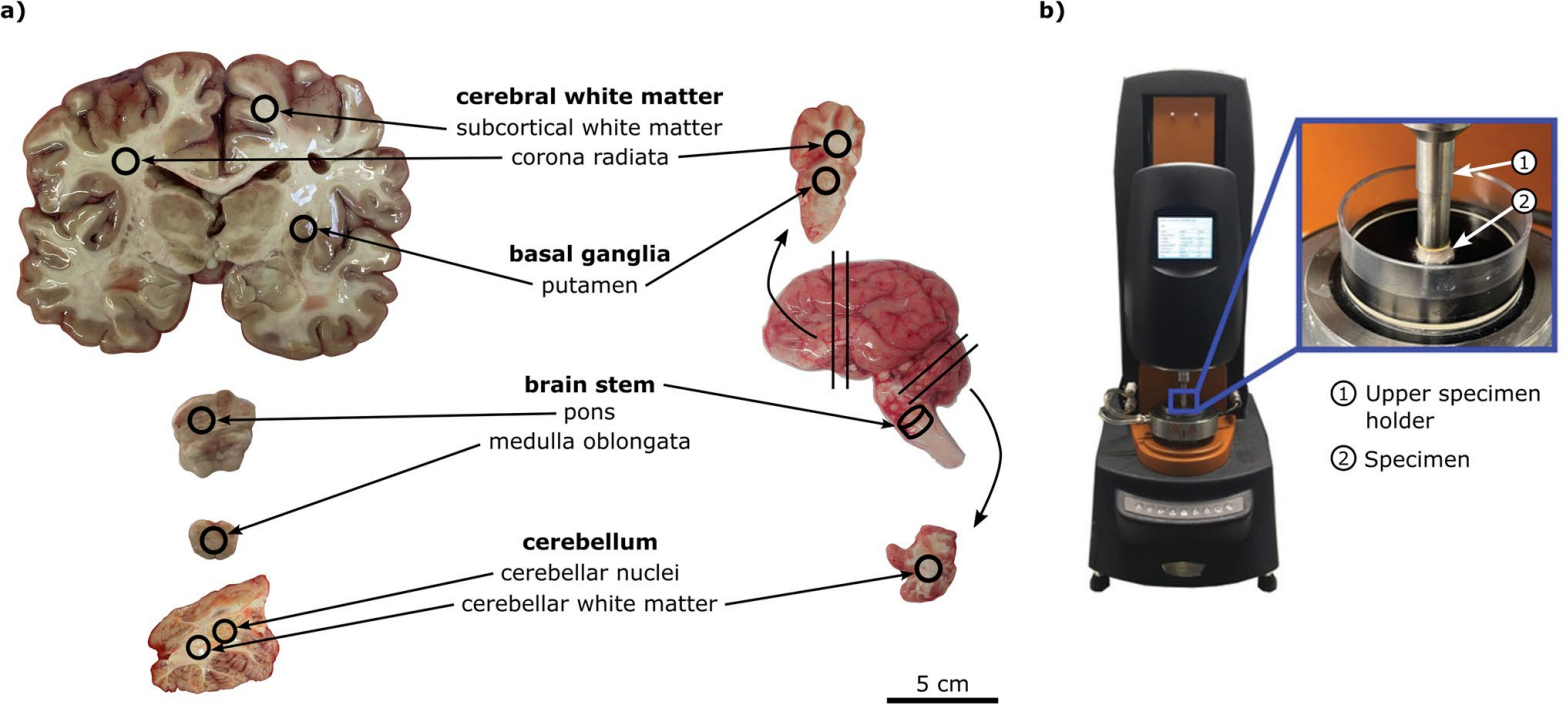
Sample extraction (**a**) and experimental setup (**b**). Cylindrical samples were punched from coronal sections of the human (**a**, left) and porcine (**a**, right) brain and then mounted to a rheometer (**b**). Photos of the porcine brain are adapted from Reiter et al. (2021), the photo of the experimental setup from Reiter et al. (2025)

We collected porcine brains from a local butcher shortly after butchering. The brains are usually from female, 4-month-old pigs, but variations are possible. Due to the butcher’s meat disassembly process, the brains were halved and disconnected from the spinal cord. The brain stem and cerebellum were included only in a few exceptions and often completely separated or partially torn off the cerebrum. We received the brain halves in a plastic bag that we kept in an airtight container during transport to prevent dehydration. The exact slaughtering time for the pigs is not known, but we completed the experiments on the day of pickup, which leads to a postmortem interval of maximum 14 h, assuming that the brains were taken from the disassembly line after the opening time of the store. In total, we used 29 brain halves for testing. Since we started the experiments right after collecting the brains, we did not immerse them in fluid, but we kept the brain halves intact for storage at $4\,^{\circ }\hbox {C}$. We prepared a coronal slice of the cerebrum (at the height of the olfactory tubercle) or a section of the cerebellum or brain stem right before testing. Slicing directions are shown in Fig. 1. We extracted one sample per region per brain half, i.e., when a brain half contained all four regions, we extracted four samples from it. Most brain halves did not contain the brain stem and cerebellum. From those, we extracted one sample from the putamen and one from the corona radiata.

### Sample preparation

We used a circular punch to extract cylindrical samples of 8 mm diameter. Due to the size difference between porcine and human brains and the different ratio of brain size to sample size, it was not possible to extract perfectly equivalent samples from both brains, i.e., the regional classification for porcine brains is more coarse than for human brains (see Fig. 1). For porcine brains, we could extract samples of the required dimensions from the corona radiata/cerebral white matter, putamen, cerebellar white matter, and brain stem, but we could not test the corona radiata and subcortical white matter separately, and it was not possible to distinguish clearly between the pons and medulla oblongata. Further, the porcine cerebellum is just large enough to extract one sample from the cerebellar white matter. Our samples from the porcine cerebellum thus presumably contain the cerebellar nuclei. Therefore, we compare the porcine corona radiata, cerebellar white matter, and brain stem to two human regions each (porcine corona radiata vs. human corona radiata and human subcortical white matter, porcine cerebellar white matter vs. human cerebellar white matter and human cerebellar nuclei, porcine brain stem vs. human medulla oblongata and human pons).

Each sample was prepared right before being tested mechanically to ensure that the samples only experienced minimal gravity-induced deformation before being attached to the rheometer. To reduce friction between the punch and the sample during extraction, we either directly punched samples out of immersed coronal slices (human brain samples) or pipetted Dulbecco’s Phosphate Buffered Saline Solution (DPBS) into the upper opening of the punch after punching to help the sample slide out (porcine brain samples). We aimed for sample heights of approximately 5 mm. Human tissue cylinders were often higher due to the thicker coronal slices, and were then carefully shortened with a scalpel. Still, some of the sample heights recorded by the rheometer after attaching the samples were lower than 4 mm or higher than 6 mm. These samples were excluded in this study to limit the variations in applied strain rates during velocity-controlled testing (see Sect. 2.4).

### Mechanical testing

For the mechanical characterization of brain samples, we used Discovery HR-3 and HR-30 rheometers from TA Instruments (New Castle, Delaware, USA). The measurement resolution and sensitivity specifications for both devices are the same except for the minimum torque, which is lower in the newer HR-30. Details are given in Supplementary Table 1. The default gap closure settings for the HR-30 differed from the HR-3. To ensure that measurements from the newer HR-30 are comparable to the previously bought HR-3, we adjusted the gap closure settings in the HR-30 to match those of the HR-3. Apart from the default gap closure settings, we noticed no differences between the loading profiles of the two devices.

We used the rheometers to measure the stress response of brain tissue under large-strain compression, tension, and torsional shear, since these loading modes can be consecutively performed on the same samples in our measurement device.

To prepare the rheometer for testing, we first attached sandpaper to the upper and lower sample holder with superglue (Pattex Ultra Gel) to improve adhesion of the brain samples. Then, we calibrated the instrument (inertia, friction, and zero gap height) for the current specimen holders with the sandpaper attached. Calibrating the gap height with the sandpaper attached later enables us to assume that the gap height measured by the rheometer once the sample is fixed equals the height of the sample (neglecting the thin layers of glue).

We fixed the specimens to the upper specimen holder with the same superglue as used for fixing the sandpaper, applied superglue to the lower specimen holder, and then carefully lowered the upper specimen holder to close the gap, i.e., establish full contact between the sample and the glue on the lower sample holder, without deforming the sample. We waited 30 to 60 s to let the glue dry and then added DPBS to immerse the specimen and keep it hydrated during the experiment. The lower sample holder was heated to $37\,^{\circ }\hbox {C}$ before the start of the experiments.

The testing protocol is shown in Table 1. We first applied three compression–tension cycles with minimum and maximum stretches of $\lambda = h / H = [H + \Delta z] / H = 0.85$ and $\lambda = 1.15$ and a loading velocity of 40 µm/s. *H* denotes the initial specimen height, i.e., the initial gap height measured by the rheometer, *h* is the current gap height, and $\Delta z$ is the displacement in the direction of loading. For velocity-controlled experiments, the resulting strain rate depends on the sample height. With a mean sample height of $\bar{H} = 5167 \pm 963$ µm, the mean applied strain rate was $\dot{\varepsilon }= 0.0080 \pm 0.0016\ 1/\text {s}$. After cyclic compression-tension, we performed compression and tension relaxation tests at the same maximum stretches with a loading velocity of 100 µm/s ($\dot{\varepsilon }= 0.0201 \pm 0.0040\ 1/\text {s}$) and a holding period of 300 s. Afterward, we performed two sets of cyclic torsional shear tests with maximum shear strains of $\gamma = 0.15$ (angular frequency $\omega = 0.333$, resulting shear rate $\dot{\gamma }= 0.2671 \pm 0.0527\ 1/\text {s}$) and $\gamma = 0.3$ ($\omega = 0.167$, $\dot{\gamma }= 0.1340 \pm 0.0264\ 1/\text {s}$), respectively. The cyclic torsional shear tests were followed by a torsional shear relaxation test at $\gamma = 0.3$. For compression and tension tests, we recorded the corresponding force $f_z$ and determined the nominal stress as $P=f_z/\pi R^{2}$, where *R* is the radius of the undeformed specimen. For torsional shear tests, we recorded the corresponding torque *t* and determined the torsional shear stress as $\tau =2t/\pi R^{3}$.

After completion of the mechanical test, the samples were carefully removed with a scalpel before cleaning the sample holders and preparing them for the next sample. Human brain samples were transferred to formalin (4% formaldehyde) containers for histological analysis, as described in detail in Reiter et al. (2025).

**Table 1:** Testing protocol

1	Cyclic compression/tension	3 cycles; up to 15% strain
2	Compression relaxation	at 15% strain; hold time = 300 s
3	Tension relaxation	at 15% strain; hold time = 300 s
4	Cyclic torsional shear	3 cycles; up to an amount of shear of $\gamma =0.15$
5	Cyclic torsional shear	3 cycles; up to an amount of shear of $\gamma =0.3$
6	Torsional shear relaxation	at $\gamma =0.3$; hold time = 300 s

### Data analysis

Data analysis was performed with MATLAB R2022b. Due to the extremely soft nature of the tissue, not all measured data were physically meaningful. Therefore, we first analyzed all raw force and gap datasets and excluded stress relaxation data when stresses increased during the relaxation period, and torsional shear data when the upper sample holder had rotated before torsional shear tests started. A more detailed description of data exclusion criteria, including examples of excluded curves, is given in Reiter et al. (2025).

Before averaging, we interpolated the experimentally measured stress data of the individual specimens to a common stretch (cyclic compression/ tension), strain (cyclic torsional shear), or time (stress relaxation) vector. Then, we grouped samples according to species and regions and determined the mean for each group. In addition to the mean, we also determined the median for each group to account for potential outliers that may skew the mean tissue response.

For statistical comparison of the unconditioned cyclic loading responses, we compared stress maxima of the first cycle in compression, tension, and torsional shear. For stress relaxation tests, we compared the degree of relaxation after 5 min.

We performed multiple comparisons with one-way ANOVA tests when all samples were normally distributed and Kruskal–Wallis tests otherwise. As a test for normal distribution, we used the Anderson-Darling test. For the post-hoc test, we used the MATLAB function multcompare.m. We consider differences to be statistically significant for values of $p < 0.05$. Statistical analysis only gives meaningful results with sufficiently large sample sizes. However, especially for tension relaxation tests, our resulting sample numbers are as low as $n=3$ for some brain regions. To ensure interpretable results, we only performed statistical analysis when each of the compared groups had a sample size of at least $n=5$.

To assess the effect size, we determined Cohen’s *d* using the MATLAB function meanEffectSize.m. As in previously published studies on mechanical testing of brain tissue (Hoppstädter et al. 2022; Reiter et al. 2025), we define the effect to be weak for $d\ge 0.1$, medium for $d\ge 0.3$ and strong for $d\ge 0.5$.

In addition to mechanical data, we qualitatively analyzed hematoxylin-eosin sections of the measured human brain samples to assess aging-related changes that may have influenced their mechanical behavior. The preparation of sections and the hematoxylin-eosin staining protocol are described in Reiter et al. (2025).

## Results

### Differences noted during handling

**Fig. 2:**
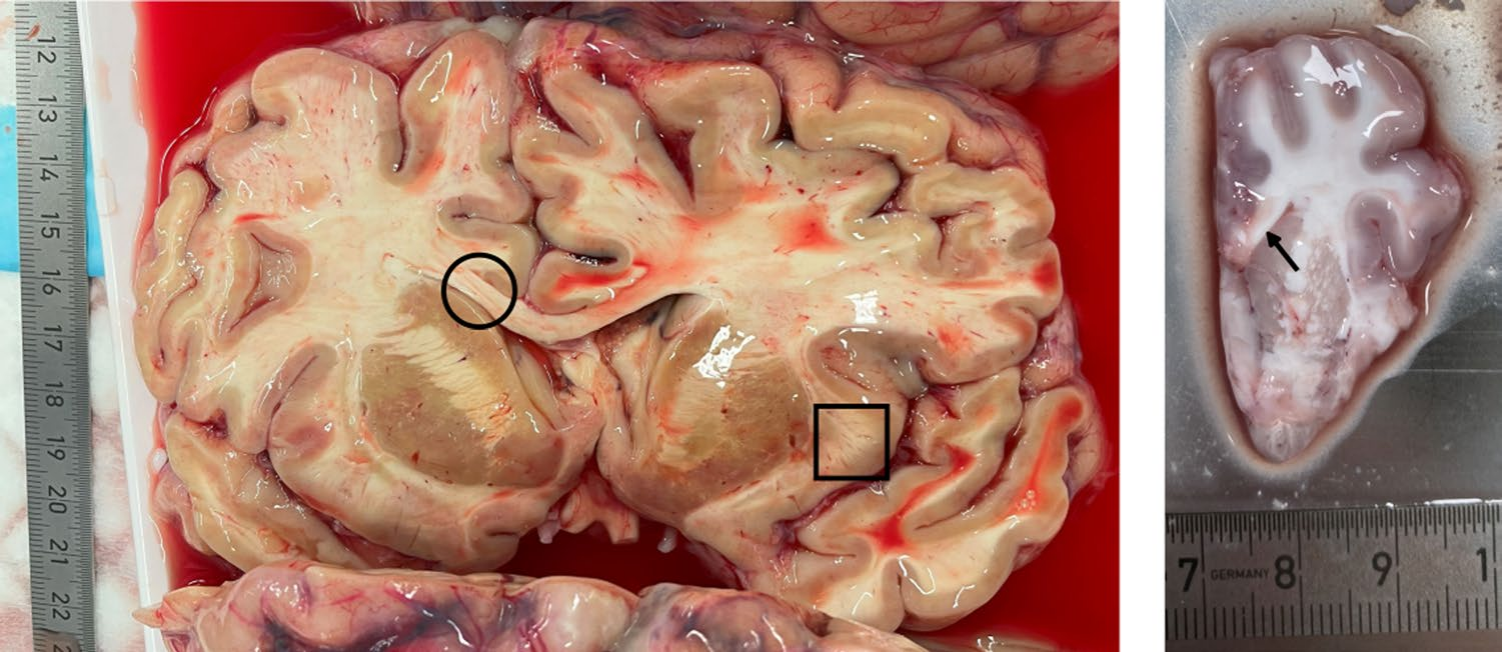
Comparison of a freshly cut human (left) and porcine (right) brain slice. The surface of the human slice is less smooth with a structured texture visible in the corpus callosum (black circle) and gaps in the subcortical white matter (black square). The slice of a porcine hemisphere appears smooth with no textured surface, even in the corpus callosum (black arrow)

We noticed a few differences between porcine and human brain tissue during handling, as described in the following. First, cut surfaces of porcine brains slices were smoother, while those from human brains sometimes had visible holes in the white matter and gaps between fiber tracts, especially in the corpus callosum (see Fig. 2). This could be related to larger blood vessel diameters (and thus visible gaps when the blood vessels collapse after blood has drained out), age-dependent changes in humans, such as arteriolosclerosis and enlarged perivascular spaces, or longer postmortem intervals, during which thinner connections between fiber tracts may have degraded. Second, porcine brain tissue seemed to be more ductile and more sticky than human brain tissue. Therefore, human tissue was easier to cut, which resulted in cylinders with sharper, better defined edges. Third, in human brains, white matter and gray matter were more clearly separated across the entire brain, while in porcine brains, it was difficult to identify the cerebellar nuclei and the gray matter areas in the brain stem.

### Age-related histological changes in human body donor brains

**Fig. 3:**
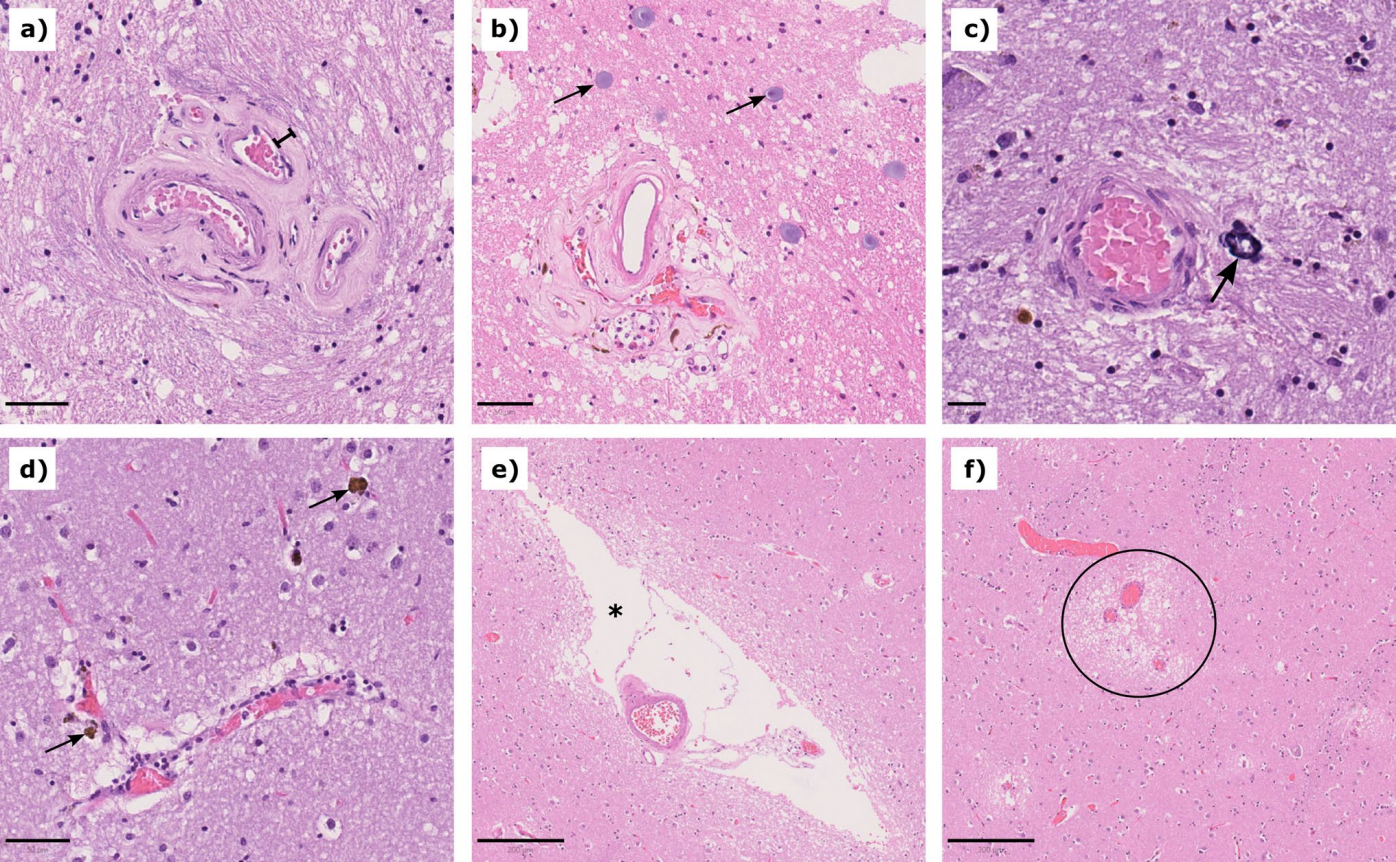
Age-related changes in human brains. Subfigures **a** and **b** show arteriolosclerosis in the corona radiata of a 92 year old male donor (**a**; the ruler symbol indicates the thickened vessel wall) and the medulla oblongata of a 78 year old male donor (**b**). In **b**, there are also corpora amylacea (arrows). Subfigure **c** shows calcification of a small vessel in the putamen of the 92 year old donor (arrow). In **d**, arrows indicate hemosiderin accumulations in the putamen of a 68 year old male donor. Subfigures **e** and **f** show the putamen of a 65 year old male donor. In **e**, an enlarged perivascular space is shown (white space marked with asterisk), and in **f**, perivascular edema are shown (black circle). Scale bars in **a**, **b**, and **d** $=50$µm, scale bar in **c** $=20$µm, scale bars in **e** and **f** $=200$µm

The human brains used in this study are from body donors that were between 62 and 92 years old. Aging has been shown to be associated with changes of brain microstructure (Schilling et al. 2022; Callaghan et al. 2014; Blinkouskaya et al. 2021), which may contribute to the observations during tissue handling described in the previous subsection. These can include arteriolosclerosis, calcification of blood vessels, hemosiderin from old bleedings, enlarged perivascular spaces, plaques, tangles, corpora amylacea, loss of myelinated axons, and changes in the extracellular matrix (Blinkouskaya et al. 2021; Blevins et al. 2021; Yachnis 2019; Marner et al. 2003; Lehner et al. 2024). The listed changes do not occur uniformly across the brain and they are not always associated with a neurological disease, i.e., certain amounts of the listed changes can occur in healthy individuals, while changes above a certain threshold would be considered pathological. In the samples that were included in this study, we mainly detected arteriolosclerosis and corpora amylacea in hematoxylin-eosin stains, as shown in Fig. 3a–b. In some samples, we saw hemosiderin, calcification of small vessels, and enlarged perivascular spaces, as shown in Fig. 3c–e. We further noticed perivascular edema in some samples (see Fig. 3f), which may have formed after death.

### Cyclic loading response

**Fig. 4:**
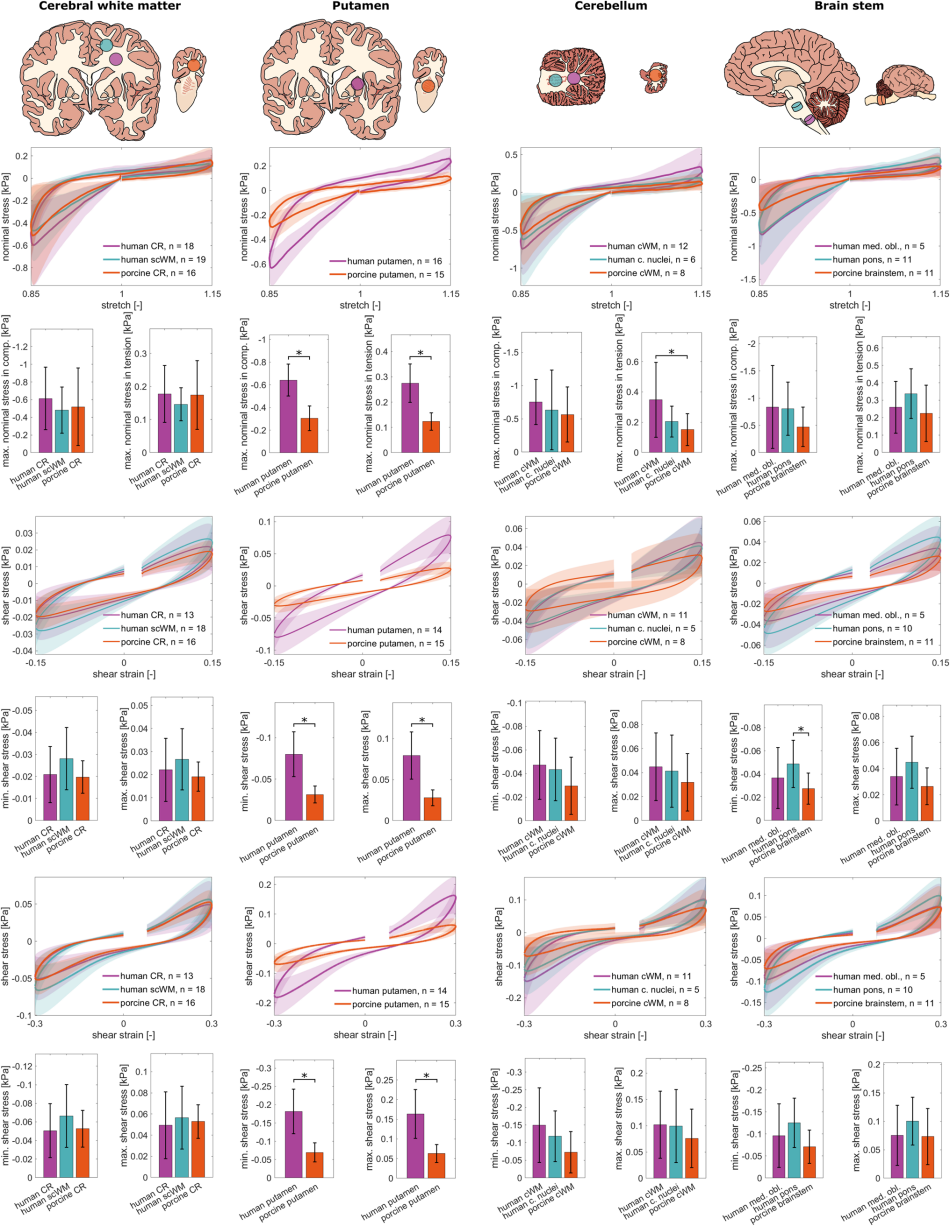
Multimodal mechanical response of different human and porcine brain regions. Averaged cyclic loading response in compression-tension (first row), cyclic shear up to a maximum of 15% shear strain (third row), and shear up to a maximum of 30% shear strain (fifth row) with corresponding minimum and maximum stresses (mean ± SD) shown below the respective stress/stretch and stress/strain curves. In torsional shear tests, there are gaps at the beginning of the first load cycle because the torque sensor starts recording the torque in the sample with a time lag due to inertia. Asterisks denote statistically significant differences, *n* denotes the sample size

Figure 4 shows the averaged stress-stretch/stress–strain response of human and porcine tissue samples from the cerebral white matter, putamen, cerebellar white matter, and brain stem during cyclic compression-tension and cyclic torsional shear tests. The average response during the first cycle, representing the unconditioned material response, is plotted together with the standard deviation. Values of Cohen’s *d* for the comparisons of stress maxima are shown in Table 2. The corresponding median curves are shown in Supplementary Figure 4.

All three loading cycles, as well as the average response during the third cycle, representing the conditioned material response, are shown in Supplementary Figures 1 and 2. Both human and porcine brain samples from all regions show the typical mechanical characteristics that have previously been described for brain tissue (Budday et al. 2019), such as hysteresis, compression-tension asymmetry, and conditioning. Conditioning describes a reversible softening (Budday et al. 2019) of the tissue response during repetitive loading. Here, we observe conditioning in all tested regions from both human and porcine brains.

In Fig. 4, it can be seen that the porcine corona radiata most closely resembles its human counterparts with no statistically significant differences. Absolute values of Cohen’s *d* between 0.03 and 0.28 indicate a weak effect of differences between human and porcine corona radiata mechanics. Differences between the human subcortical white matter and the porcine corona radiata have a weak to strong effect depending on the loading mode, with absolute values of Cohen’s *d* ranging from 0.10 to 0.71. The median curves of the three groups show the same trend (see Supplementary Figure 4).

The porcine putamen most strongly differs from the human putamen for all loading modes and irrespective of whether the mean or median is considered. Differences between the stress maxima measured for human and porcine putamen samples in compression, tension, and shear are statistically significant with absolute values of Cohen’s *d* between 2.13 and 2.60 indicating a strong effect.

Both the mean and median stress response of samples from the porcine cerebellum are softer than the stress response of samples from the human cerebellum, especially during torsional shear tests. Porcine cerebellum samples more closely resemble human cerebellum samples that contain the cerebellar nuclei in compression and tension. In tension, the difference between the porcine cerebellum and human cerebellar white matter is statistically significant with an effect size of $d=0.92$.

According to mean values, porcine brain stem samples are softer than samples from the human pons and medulla oblongata for all loading modes. However, the median shear stress response of the porcine brain stem closely resembles that of the human medulla oblongata. In compression and tension, the median stress response of the porcine brain stem is closer to the medulla oblongata than to the pons. Differences in mean are only statistically significant for the comparison of the human pons and porcine brain stem under cyclic shear loading up to 15% strain. The absolute values of Cohen’s *d* range from 0.04 to 0.67 for differences between the human medulla oblongata and the porcine brain stem, indicating a weak to strong effect, depending on the loading mode. For differences between the human pons and porcine brain stem, the effect is strong for all loading modes, with absolute values of Cohen’s *d* between 0.56 and 1.20.

### Stress relaxation behavior

**Fig. 5:**
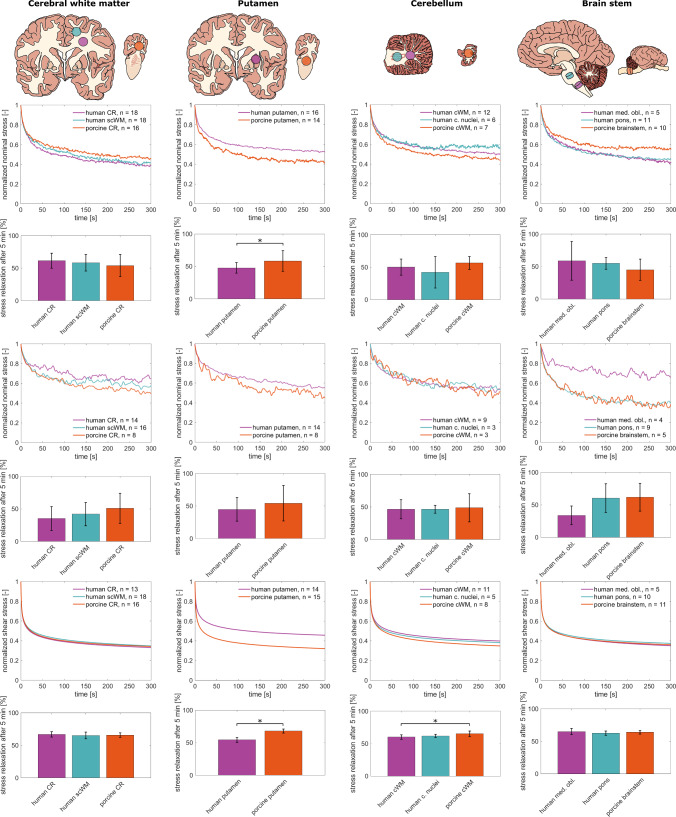
Averaged, normalized stress relaxation response of human and porcine tissue samples from the cerebral white matter, putamen, cerebellar white matter, and brain stem in compression (first row), tension (third row), and torsional shear (fifth row) with corresponding stress relaxation percentages after 5 min (mean ± SD) shown below the respective relaxation curves. Asterisks denote statistically significant differences. For the cerebellum and brain stem, statistical analysis was not done for tension relaxation tests due to the small sample sizes ($n<5$)

Figure 5 shows the mean normalized stress relaxation behavior of human and porcine brain tissue in compression, tension, and shear. The compression and tension stress relaxation curves appear noisy despite being averaged because the changes of measured forces are relatively small, especially during the later holding phase. Shear relaxation curves are smooth due to the high torque sensitivity of the rheometer (see Supplementary Table 1). The median normalized stress relaxation response is shown in Supplementary Figure 5. Values of Cohen’s *d* for the comparison of the normalized stress relaxation behavior are given in Table 2. The normalized stress relaxation behavior of human and porcine brain tissue is generally similar, but absolute peak and equilibrium stress values vary, as shown in Supplementary Figure 3.

The mean curves in Fig. 5 show that the porcine corona radiata relaxes more than the human corona radiata and subcortical white matter under tension. The difference is not statistically significant and only visible for the comparison of the porcine corona radiata and human corona radiata when the median is considered, but has a strong effect with Cohen’s $d=-0.74$ for the comparison between the porcine and human corona radiata. The mean and median compression and shear relaxation behavior of the porcine corona radiata closely resembles the behavior of the human corona radiata and subcortical white matter. Corresponding absolute values of Cohen’s *d* range from 0.14 to 0.51.

The porcine putamen relaxes significantly more than the human putamen in compression (58% vs. 48%) and shear (68% vs. 54%). The associated values of Cohen’s *d* of $d=-0.83$ and $d=-3.88$, respectively, indicate a strong effect. Differences in tension relaxation are not statistically significant and have an effect size of $d=-0.42$. The same trend can be seen for the median stress relaxation response.

The porcine cerebellar white matter relaxes more in compression than the human cerebellar white matter and cerebellar nuclei (57% vs. 50% and 42%). Differences are not statistically significant and have an effect size of −0.53 for the comparison between the porcine and human cerebellar white matter, and −0.75 for the comparison between the porcine cerebellar white matter and human cerebellar nuclei. When the median is considered, no differences between the porcine and human cerebellar white matter are observed. In shear, the porcine cerebellar white matter relaxes slightly more than the human cerebellar white matter and cerebellar nuclei (65% vs. 60% and 62%). The difference in relaxation between the porcine and human cerebellar white matter is statistically significant with an effect size of $d=-1.24$. We observed minor differences in the tension relaxation behavior of the three groups. Since some of the curves recorded during tension relaxation tests were excluded because the force increased during the holding period (see Sect. 2.5), the resulting sample size for this step of the testing protocol was too small for proper statistical analyses.

The mean and median stress relaxation behavior of the porcine brain stem more closely resembles the behavior of the human pons than of the human medulla oblongata. In compression, the porcine samples relax less than the human samples (porcine brain stem: 45%, human pons: 55%, human medulla oblongata: 59%) when mean values are considered. The differences have a strong effect with values of Cohen’s $d=0.60$ and $d=0.73$, respectively, but are not statistically significant, and differences between the human pons and porcine brain stem are not visible when the median is considered. For tension relaxation tests, statistical analyses could not be performed because of insufficient sample sizes due to exclusion of unphysical data. Here, it stands out that the human medulla oblongata relaxes less in tension than the human pons and porcine brain stem, which behave similar to each other. This trend is the same for mean and median curves. The mean and median shear relaxation behavior of all three groups is almost the same. Differences in mean between the three groups are not statistically significant and have effect sizes of $d=0.28$ for the comparison between the porcine brain stem and human medulla oblongata, and $d=-0.43$ for the comparison between pons and medulla oblongata.

**Table 2:** Effect size/absolute values of Cohen’s *d* for comparisons of stress maxima and stress relaxation after 5 min

	Human CR—porcine CR	Human scWM—porcine CR	Human putamen—porcine putamen	Human cWM—porcine cWM	Human c. nuclei—porcine c. nuclei	Human medulla obl.—porcine brain stem	Human pons—porcine brain stem
Max. nominal stress in compression	−0.23	0.10	−2.60*	−0.49	−0.13	−**0.67**	−**0.74**
Max. nominal stress in tension	0.03	−0.35	**2**.47*	**0**.92*	0.49	0.21	**0**.**71**
Max. shear stress (15% strain)	0.28	**0**.**69**	**2**.36*	0.28	0.33	0.43	**1**.**04**
Min. shear stress (15% strain)	−0.11	−**0.71**	−2.35*	−**0.62**	−**0.51**	−0.48	−1.20*
Max. shear stress (30% strain)	−0.14	0.14	**2**.13*	0.41	0.36	0.04	**0**.**56**
Min. shear stress (30% strain)	0.08	−0.47	−2.36*	−**0.82**	−**0.66**	−0.47	−**1.10**
Compression relaxation after 5 min	**0**.**51**	0.28	−0.83*	−**0.53**	−**0.75**	**0**.**60**	**0**.**73**
Tension relaxation after 5 min	−**0.74**	−0.44	−0.42	n.a.	n.a	n.a.	n.a.
Shear relaxation after 5 min	0.30	−0.14	−3.88*	−1.24*	−**0.82**	0.28	−0.43

Species differences with a strong effect (Cohen’s $d\ge 0.5$) are indicated with bold print. For maximum stresses in compression and minimum stresses in shear, a negative value of Cohen’s *d* indicates that the porcine samples were softer than the corresponding human samples. For maximum stresses in tension and maximum stresses in shear, positive values of Cohen’s *d* indicate a softer response of porcine tissue. For stress relaxation tests, a negative value of Cohen’s *d* indicates that the porcine samples relaxed more on average. Asterisks denote statistical differences as also shown in Figs. 4 and 5

## Discussion

Our results show that human and porcine brain tissue show the same general qualitative characteristics, such as stress relaxation, hysteresis, and compression-tension asymmetry. We note that our results and conclusions are valid for moderate strain rates ($\dot{\varepsilon }= 0.0080 \ 1/\text {s}$ and $\dot{\varepsilon }= 0.0201 \ 1/\text {s}$ for compression/ tension tests, $\dot{\gamma }= 0.2671 \ 1/\text {s}$ and $\dot{\gamma }= 0.1340 \ 1/\text {s}$ in torsional shear tests) that are applicable e.g., to surgical procedures. While we have not specifically assessed strain rate dependence in this study, we expect that the species dependent trends we observed are transferable to different strain rates, since both human and porcine brain tissue have been shown to stiffen with higher strain rates (Budday et al. 2019).

During cyclic loading tests, the porcine putamen was consistently softer than the human putamen in all loading modes, while tissue from the cerebral white matter behaved similarly in both species. For samples from the cerebellum and brain stem, trends differ depending on whether mean or median curves are considered, which is mainly due to differences between mean and median curves of human regions. This indicates that the human samples contain outliers that affect the comparison of the two species. In addition, the stiffness ratios between regions were different for the different species. This is especially relevant for the putamen, which is stiffer than cerebral white matter in the human brain, while the porcine putamen is softer than porcine white matter. These differences could be caused by differences in tissue structure: the samples extracted from the human putamen were macroscopically homogeneous gray matter samples, whereas samples from the porcine putamen consisted of visible layers of gray and white matter.

The stress relaxation behavior was similar in both species. This observation agrees with a previous study by MacManus et al. (2020). However, there is one exception, the putamen, where we observed differences in the relaxation behavior between the two species for all loading modes. The overall similarity in the relaxation behavior of both species means that porcine brain tissue is a suitable surrogate for human tissue when investigating the viscoelastic behavior of brain tissue. We have previously used porcine tissue to derive a microstructure-based tissue stress relaxation time (Reiter et al. 2021) that we have later successfully used to fit human tissue data (Reiter et al. 2023a). Other applications, in which porcine brain tissue has proven to be a suitable surrogate for human tissue, are studies that aim to establish testing procedures and better understand the influence of sample preparation and postmortem time (Garo et al. 2007), studies that aim to gain insights into multiscale tissue mechanics (Begonia et al. 2010; Reiter et al. 2023b) that are likely transferrable between species, studies that look at age-dependent trends and thus require samples of a defined age range (Prange and Margulies 2002; Hoppstädter et al. 2024), or studies that involve mechanical impacts on the living brain (Browne et al. 2011; Cullen et al. 2016; Wolf et al. 2017).

For human brains, a finer distinction of regions was possible due to the larger brain size. For example, there are microstructural differences between the central and subcortical cerebral white matter in the human brain, i.e., axons are arranged differently in both regions, as we have shown in Reiter et al. (2025). The mechanical effect of such differences could not be investigated in the porcine brain with our setup due to the required sample dimensions. When experimental data for a detailed "mechanical atlas" of the brain are required, for example for full brain simulations of surgical interventions (Griffiths et al. 2023), human tissue may thus be more suitable (depending on the testing method and required sample dimensions). We have previously characterized 19 regions of the human brain in compression, tension, and torsional shear (Hinrichsen et al. 2023), which would not be possible for porcine brains, since most of the characterized regions are too small in the porcine brain, as can be seen in an atlas of the pig brain published by Félix et al. (1999). Still, such a refined regional characterization of porcine brain tissue could be possible with setups that either work with smaller samples or that test on a different scales, e.g., micro or nanoindentation (Elkin et al. 2011; MacManus et al. 2017, 2020; Sundaresh et al. 2021).

In addition to anatomical differences between human and porcine brains (and the brain size dependent differences in extracted samples), we also observed age-related changes in human brains that may have had an influence on the mechanical response of the tested human tissue. Arteriolosclerosis (a thickening and stiffening of blood vessel walls) and calcification of blood vessels are common in elderly people and could lead to an overall stiffer tissue response. Hemosiderin accumulations from past bleedings have been shown to contain ferritin (Richter 1958) and may thus have different mechanical properties than the surrounding tissue, but since the accumulations are rather small compared to the size of the tested specimens, we assume that their overall effect on tissue mechanics may be limited. Perivascular spaces and edema may soften the tissue. Corpora amylacea are granular bodies formed by polyglucosan aggregates and are considered to be containers that actively remove waste products (Riba et al. 2019). Their number increases with age. To the best of the authors’ knowledge, the mechanical properties of corpora amylacea have not been investigated yet. Results from magnetic resonance elastography studies suggest that human brain tissue softens with age (Sack et al. 2009, 2011; Hiscox et al. 2018). However, stiffness trends seen in magnetic resonance elastography do not transfer directly to multimodal biomechanical testing (Bertalan et al. 2023).

In addition to reporting the statistical significance, we have used the effect size to quantify the difference between the mechanical properties of porcine and human brain tissue and to evaluate their practical significance. For the comparison of porcine and human cerebellar white matter in cyclic shear, and the comparison of porcine brain stem and human medulla oblongata and pons in cyclic compression and compression relaxation, we found a large effect of differences in mean values, but no statistical significance. In the case of the cerebellar white matter, differences between the species are also visible for the median curves. Here, the large effect size of the mean comparison may indicate that the observed differences are still important to consider. In the case of the brain stem samples, differences in cyclic compression and compression relaxation are less clear for the median curves. Here, the lack of statistical significance could also be attributed to low sample sizes, which are typical for biomechanical studies on human tissue, and which are associated with a higher influence of outliers on the mean values. In a previous study, we have shown that considering regional stiffness variations in finite element simulations influences the accuracy of the results, even if those regional differences were previously found not to be statistically significant (Griffiths et al. 2023; Hinrichsen et al. 2023).

Our experiments showed similarly high standard deviations for porcine and human brain tissue, but differences between mean and median values were larger in human samples. This indicates that stark outliers may be more common in human tissue samples, but variations in tissue properties occur in both species and are thus probably not merely caused by pathological or age-related changes in the microstructure of humans, as we have previously hypothesized (Reiter et al. 2025), or by large differences in postmortem intervals (in the range of days) at the time of testing, as hypothesized by Chatelin et al. (2012). For porcine brain tissue, age differences are minor and postmortem interval differences do not exceed the range of 14 h, but additional uncertainties that could lead to the high standard deviations are differences in how the brains were handled at the slaughterhouse, including differences in storage time and temperature before pick up. The standard deviations were smaller in the putamen than in the other tested regions. In this region, we could consistently extract samples from the same location since, in the human brain, the caudal part of the human putamen is just large enough to obtain a single sample, and for the porcine brain, we could use the olfactory tubercle as an anatomical landmark to place the cuts for the respective coronal section, where the putamen is again just large enough to extract one sample. In addition, the consistency of the putamen, especially in the human brain, made it easier to cut than the other brain regions. This could indicate that inaccuracies in the exact sampling location, the corresponding microstructure, or the sample geometry, i.e., deviations from a perfectly cylindrical shape, may be linked to high standard deviations. We aimed to mount the samples without applying any pre-stretch. However, stronger deviations from the intended cylindrical shape make it more difficult to find a deformation-free contact point between the sample and the rheometer, so that those samples may have been slightly pre-compressed.

An important limitation of the present study is that human and porcine samples were tested at different postmortem intervals because human tissue became available for testing only later. Porcine samples were tested on the day of butchering, leading to estimated postmortem intervals of up to 14 h, whereas for human samples, postmortem intervals at the beginning of the experiment ranged from 20 to 97 h and experiments were on average performed over a course of 41 h. To slow down autolytic changes of the postmortem human tissue, brain slices were stored in artificial cerebrospinal fluid during this time. Previous studies have shown changes in tissue mechanics with death and in the first hour postmortem (Weickenmeier et al. 2018), no changes in the range from 2,5 to 6 h postmortem (Garo et al. 2007) (relevant for animal tissue and surgically resected tissue), an increase in stiffness in the range from 6 to 10 h postmortem (Garo et al. 2007) (relevant for experiments on animal tissue with longer testing durations), and no changes in the range of days (Budday et al. 2015; Forte et al. 2017) (relevant for human tissue from body donations and autopsies). Zwirner et al. found differences between day 0 and days 1 to 4, but no reliable trends after day 1 (Zwirner et al. 2023).

## Conclusion

To assess whether porcine brain tissue can be used as a surrogate for human brain tissue in multimodal mechanical testing, we have compared the mechanical response of human and porcine brain tissue of different anatomical regions during both cyclic loading and stress relaxation tests in compression, tension, and torsional shear. Human and porcine brain tissue generally showed a similar behavior especially in the cerebral white matter, but there were marked differences in the putamen. The overall similarity between the two species makes porcine brain tissue an accessible alternative for human brain tissue especially for studies that aim to gain more insights into general brain tissue mechanics or that have specific requirements regarding sample number, age, or other conditions which cannot be met with postmortem human tissue. However, since the porcine brain is smaller and therefore not all regions are large enough to extract samples for multimodal testing and since regional trends in the porcine brain may be different from those in the human brain, human brain tissue should preferably be used for experiments that provide data for parameter identification and full brain simulations.

## Supplementary Information

Below is the link to the electronic supplementary material.

Supplementary file 1 (pdf 22713 KB)

## Data Availability

The mechanical data analyzed during the current study are available from the corresponding author on reasonable request.
