# Supplementary material for: Mechanical characterization of human versus porcine brain tissue under large strains
Source: Biomech Model Mechanobiol. 2026 Mar 5;25(2):26. doi: 10.1007/s10237-025-02040-8 (PMC12963166; doi:10.1007/s10237-025-02040-8)
Supplement: Supplementary file 1 — (pdf 22713 KB) [file 10237_2025_2040_MOESM1_ESM.pdf]

Supplementary information for: Mechanical characterization of human versus porcine brain tissue under large strains

Nina Reiter[1], Sarah Nistler[1], Lucas Hoffmann[2], Lars Bräuer[3], Friedrich Paulsen[3], Silvia Budday[1*]

[1]Institute of Continuum Mechanics and Biomechanics, Friedrich-Alexander-Universität Erlangen-Nürnberg (FAU), Dr.-Mack-Str. 81, Fürth, 90762, Germany.
[2]Department of Neuropathology, Universitätsklinikum Erlangen, Friedrich-Alexander-Universität Erlangen-Nürnberg (FAU), Schwabachanlage 6, Erlangen, 91054, Germany.
[3]Institute of Functional and Clinical Anatomy, Friedrich-Alexander-Universität Erlangen-Nürnberg (FAU), Universitätsstr. 19, Erlangen, 91054, Germany.

*Corresponding author(s). E-mail(s): silvia.budday@fau.de;
Contributing authors: nina.reiter@fau.de; sarah.nistler@tuwien.ac.at; lucas.hoffmann@uk-erlangen.de; lars.braeuer@fau.de; friedrich.paulsen@fau.de;

Resolution and sensitivity specifications of the used rheometers

Supplementary Table 1 Specifications of Discovery HR rheometers.

	**HR-3**	**HR-30**
Torque resolution	0.05 nNm	0.05 nNm
Minimum torque (oscillation)	0.5 nNm	0.3 nNm
Minimum torque (steady shear)	5 nNm	1 nNm
Displacement resolution	2 nrad	2 nrad
Normal force resolution	0.5 mN	0.5 mN
Normal force sensitivity	0.005 N	0.005 N
Gap position resolution	20 nm	20 nm

Mean cyclic stress response of human and porcine brain tissue during all three loading cycles

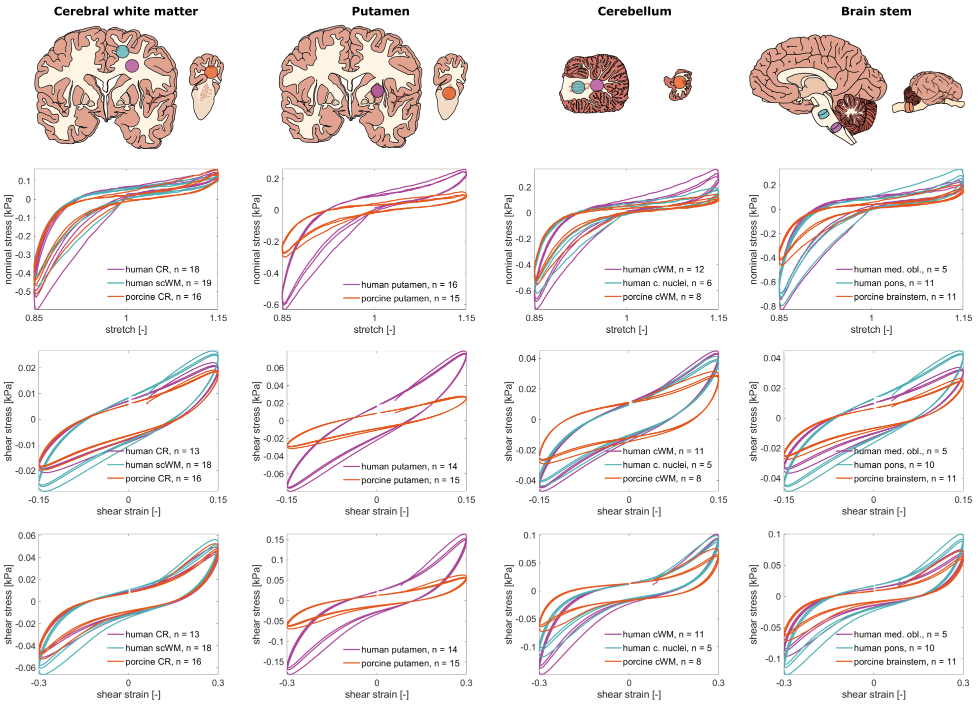

Supplementary Figure 1 Mechanical response of different human and porcine brain regions during cyclic loading tests. Averaged cyclic loading response in compression-tension (first row), cyclic shear up to a maximum of 15% shear strain (second row), and shear up to a maximum of 30% shear strain (third row).

Conditioned stress response (third loading cycle) of human and porcine brain tissue

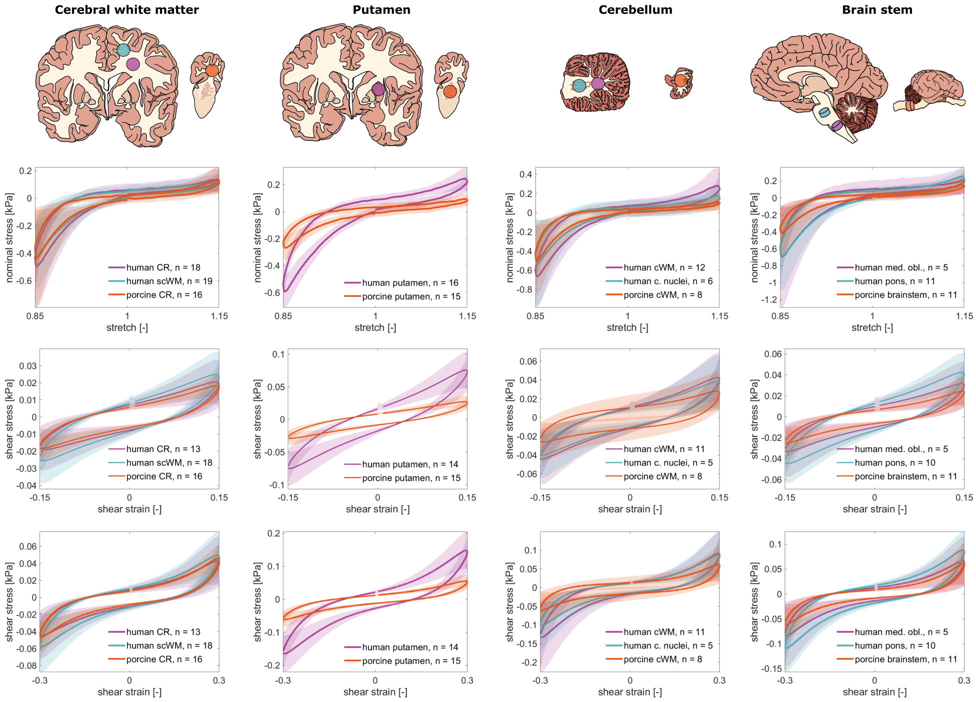

Supplementary Figure 2 Conditioned mechanical response of different human and porcine brain regions. Averaged cyclic loading response of the third cycle of compression-tension (first row), cyclic shear up to a maximum of 15% shear strain (second row), and shear up to a maximum of 30% shear strain (third row).

Mean absolute stress response of human and porcine brain tissue during stress relaxation tests

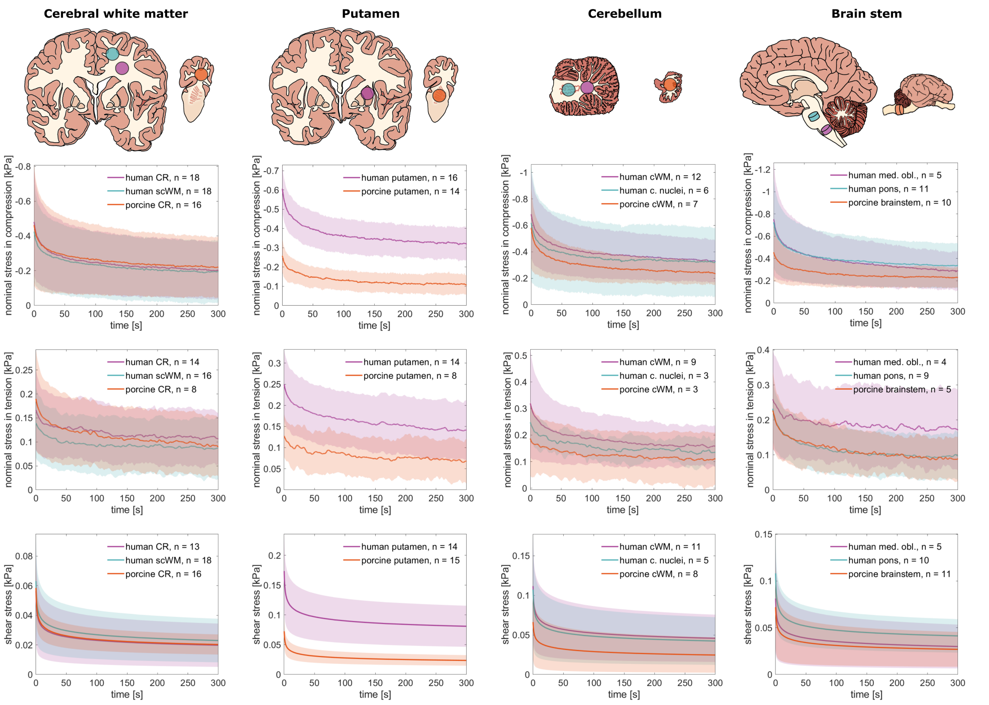

Supplementary Figure 3 Averaged stress relaxation response with standard deviation of human and porcine tissue samples from the cerebral white matter, putamen, cerebellar white matter, and brain stem in compression (first row), tension (second row), and torsional shear (third row).

Median cyclic stress response of human and porcine brain tissue in compression, tension, and torsional shear

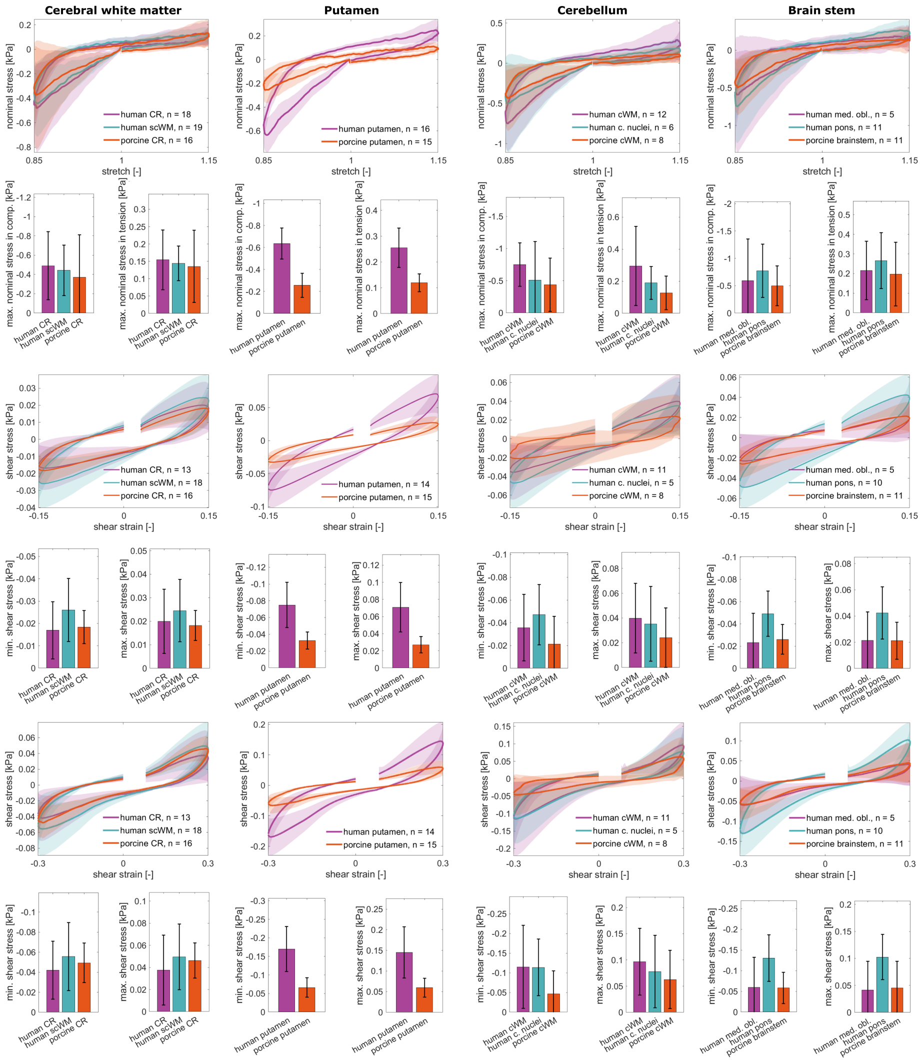

Supplementary Figure 4 Multimodal mechanical response of different human and porcine brain regions. Median cyclic loading response in compression-tension (first row), cyclic shear up to a maximum of 15% shear strain (third row), and shear up to a maximum of 30% shear strain (fifth row) with corresponding minimum and maximum stresses (median ± SD) shown below the respective stress/stretch and stress/strain curves.

Median stress response of human and porcine brain tissue during stress relaxation tests

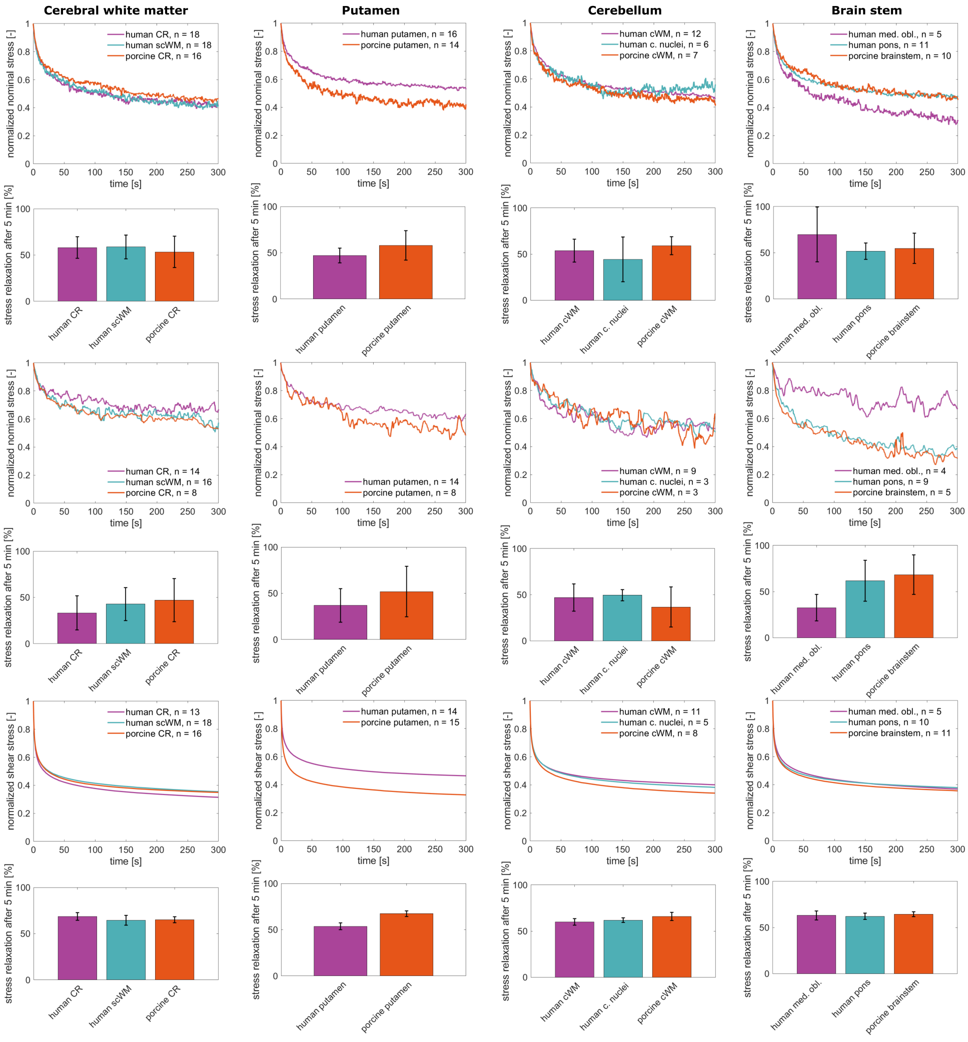

Supplementary Figure 5 Median normalized stress relaxation response of human and porcine tissue samples from the cerebral white matter, putamen, cerebellar white matter, and brain stem in compression (first row), tension (third row), and torsional shear (fifth row) with corresponding stress relaxation percentages after 5 min (median ± SD) shown below the respective relaxation curves.
